# Book Reviews

**Published:** 1801-04

**Authors:** 


The number of popular Treatifes on the Cow-pox, in all European languages, increafes fo faft, that we can do little more than detail their titles.

*Familiar Observations on the Inoculation of the Cow-pox, as now very generally introduced in Great Britain, and several parts of the Continent; with a View to the final Extirpation of Small-pox.* By Alex. Herman Mac Donald, M. D. 4to. pp. 50. Hamburgh, 1800.

We have no hefitation in recommending this correct and impartial work to the attention of our countrymen, though compiled from our own materials, and publifhed at a confiderable diftance from the fource of the moft recent information.

---

*Evidences of the Utility of Vaccine Inoculation; intended for the Information of Parents.* Dedicated, by permiffion, to the Right Hon. Lord Somerville. By Thomas Creaser, Member of the Royal College of Surgeons. 12mo. pp. 36, price 1s. Bath, 1800, Crutwell.

The Author, who appears to have directed his attention to this fubject from its firft eftablifhment as a branch of medical fcience, fays,

"Little more than two years have elapfed, fince Dr. Jenner announced to the public, the fingularly curious and beneficial effects of Vaccine Inoculation. His conclufions were formed from experiments fuggefted by facts founded on the unerring bafis of experience. From this fource we have derived the application of bark, mercury, opium, and almoft all the moft powerful agents of medical dical fcience. Extenfive and impartial trials of the truth of Dr. Jenner's pofitions were immediately inftituted, and they have been profecuted with avidity by men of the firft talents, independent of concert or co-operation. The refult is, that although the Inoculation of the Cow-pox is one of the boldeft and moft direct innovations on preceding practice, and, as fuch, has had to encounter all the impediments which are ufually oppofed to novelty, by the operations of fcepticifm, prejudice, and intereft; yet its afferted and almoft unparalleled advantages have been realized in their higheft extent, by a mafs of irrefiftible evidence. It is truly extraordinary, that in a meafure fo perfectly original, and which has been fo immediately fubmitted to the teft of extenfive experiment, fubfequent inveftigation fhould have tended merely to confirm the pofitions of the original propofer. It is a law of the human mind to prefer good to evil, provided it be competently informed of their difference, and that it is not under the influence of erroneous judgment or habits. Of the quantity of evidence exifting to demonftrate the highly preferable nature of the Vaccine to the Small-pox Inoculation, the public want only acceffible means of information.

"It will be neceffary previoufly to enter into the hiftory of the natural Cow-pox, as diftinguifhed from the fame difeafe when applied by inoculation, and as the origin of the latter. The cow, though in general a healthy animal, is fubject to this and a few other difeafes.

If, in a popular work, we may be allowed to digrefs a little into the pathology of animals, it may be obferved, as a curious fact, that their predifpofitions to difeafe are of the fpecific kind. The horfe is liable to broken-wind, farcy, glanders, and greafe, exclufive of fome local difeafes, the greater number of which do not arife from original or natural caufes, but from the unnatural and violent fituations to which he is expofed, whilft adminiftering to the utility and luxury of man. The fheep is difpofed to the difeafe termed the rot, and to hydatids of the brain. The dog to its peculiar affection called the diftemper, to mange, and hydrophobia. The cow is particularly liable to the difeafe of which we are treating, and to the yellows, which are fimilar to the human jaundice. Much phyfiological confideration arifes out of thefe obfervations; not only the morbid fecretions of the fame animal are capable of converfion and mutual change, but alfo the fame morbid poifon, applied to different animals, feems to produce not a fimilar and fpecific difeafe, but the difeafe to which the animal, from conftitution and ftructure, is pre-difpofed. It is now confirmed, according to Dr. Jenner's original theory, that the Cow-pox originates in a morbid poifon communicated from the greafy heel of the horfe; yet this poifon does not produce the Cow-pox in the human fubject, till it has been received by, and tranfmitted from, the cow. My friend Mr. Coleman, Profeffor of the Veterinary College, has created glanders by inoculating the membrane of the noftrils of the horfe from its farcy buds, as they are termed; i. e. inflamed and ulcerated lymphatics; the ſame effect took place, *vice versa*, from glanders to farcy; and, which is truly ſingular, Dr. Jenner informs me, that dogs, inoculated with vaccine matter, acquire their peculiar affection, termed the Diſtemper. Theſe ſpeculations lead us to a vaſt field of conſideration concerning the modifications of morbid poiſons, and they teach us alſo not to regard the introduction of them as hazardous on another claſs of animals, when they become remedial alternatives."

This is a beautiful little work, and admirably calculated to anſwer the views of the enlightened Author.

---

*Memoire sur l'Inoculation de la Vaccine a Geneve*, par L. Odier, Dr. & Prof. en Medicine; 8vo. pp. 30.

This excellent pamphlet deſcribes ſeveral kinds of eruptions which may occaſionally take place during the progreſs of the Vaccine Inoculation, but not eſſentially connected with it.

---

*Essai sur l'Inoculation de la Vaccine, ou moyen de se preserver, pour toujours et sans danger de la petite Verole;* par F. Colon, M. D. 8vo. pp. 36. Paris.

That our Readers may ſee in more lights than one, the ardour with which the new Inoculation is purſued in France, we preſent them with a tranſlation of this Author's Introduction.

"However aſtoniſhing the diſcovery of the Vaccine Inoculation may appear, and although the Engliſh have enjoyed the benefits of it for upwards of two years with the greateſt ſucceſs, its benefits are, nevertheleſs, ſcarcely known in France.

"It is well known that about the commencement of the laſt autumn, M. Larochefoucault Liancourt propoſed to open a ſubſcription for defraying the expences neceſſary to make proper trials of the Vaccine Inoculation, and to decide on its preference to that of the Small-pox. This propoſition of M. Liancourt was cordially received by many benevolent individuals: In a ſhort time a fund was raiſed adequate to the object propoſed.

"A medical committee was appointed to verify the phænomena which precede, accompany, and follow the Vaccine Inoculation.

"To haſten as much as poſſible the iſſue of theſe propoſed trials, I offered my houſe at Vaugirard, where forty could be conſtantly under inoculation without the poſſibility of any extraneous influence on the progreſs of the diſeaſe.

"In thus paying my tribute to the diſcovery which at preſent engroſſes the attention of the medical world, I acquired an opportunity of obſerving its ſucceſs, and of being able to ſpeak of it with ſome degree of knowledge.

"I by no means propoſe here to enter into minute details, or to write as a member of the medical committee on the Vaccine Inoculation: My individual opinion has nothing in common with the relation in which the committee is bound to the public. They will, will, no doubt, fpeedily publifh the refult of their experience,* and will fatisfy the expectations and confidence of profeffional men and the public at large; but having inoculated the Vaccine at Paris, amidft the clamours of a party that wifhed to oppofe its propagation, I owe it to thofe perfons who have honoured me with their confidence, to lay before them the facts which have determined my conviction, that this new Inoculation merits the preference over the old.

I pretend to no more than to give a general knowledge of the Vaccine and its advantages, to the end that thofe who are not of the profeffion, as well as young phyficians who have not been able to purfue the experiments made in my houfe, might yet be able to form juft ideas, by which they will be able to confute the lies, and the abfurd clamour raifed againft this method by interefted men, or thofe who know nothing of the matter.

"I fhall endeavour, above all, to fteer equally clear of blind credulity, which adopts every thing, and that interefted and party fpirit, that would oppofe nature herfelf, as well as the progrefs of ufeful knowledge.

"It was in effect this fpirit of party, which on the firft introduction of the Vaccine, raifed the cry of *innovation,* and founded the *tocfin* of prejudice againft trials, which had for their object, a concern as important as any within the fcope of medicine.

"It required the conftancy, the fpirit of wifdom which directs the medical committee to refift thefe clamours, which are only a repetition of the attempts made in the laft century to prevent the introduction of Inoculation into France.

"There was, undoubtedly, prudence and good fenfe in retaining their judgment on a fubject of this importance, till the committee, by its report, had freed the fubject from all difficulties; but jealoufy alone, and obftinacy, were able to give birth to fufpicions on the validity of experience itfelf, and on the motives which actuated men, who, from a zeal truly laudable, make perfonal facrifices to examine and verify the trials of the Vaccine Inoculation.

"In this effay I fhall explain what the Vaccine is, the varieties of which it is fufceptible, and the advantages it poffeffes over the common Inoculation. I fhall anfwer various objections which I have heard from thofe who wifh to throw obftacles in our way; and I fhall conclude with a comparative table of the effects of the inoculated Small-pox and the Vaccine."

This fhort work performs all it promifes, and muft have produced a confiderable effect in removing the prejudices of the Author's countrymen.

---

* See page 356.

*Comparative View of the Theories and Practice of Drs. Cullen, Brown, and Darwin, in the Treatment of Fever and of Acute Rheumatism.* By H. Xavier Baeta, M. D. 8vo. pp. 55, price 1s. 6d. London, Johnſon.

Dr. Cullen's Spaſmodic Theory of Fever is familiar to every practitioner: See his Firſt Lines, § 46, &c.

Dr. Brown's Theory of Fever is thus ſtated by Dr. Baeta.

"Dr. Brown ſuppoſes the proximate cauſe of fever to conſiſt in debility (Elem. of M. par. 679) which may be either direct or indirect, according to the nature of the noxious powers which were previouſly applied to the ſyſtem. (Elem. of M. par. 681) Hence he makes two diviſions of fevers;—1ſt, Thoſe which depend on direct debility: in this he ranks ſome intermittent fevers, the typhus mitior and gravior, the plague, &c. (Elem. of M. par. 685, 6)—2d, Thoſe which depend on indirect debility: under which diviſion he ranks ſome intermittents, and ſome continued fevers, occaſioned by drunkenneſs, the confluent ſmall-pox, &c. (Elem. of M. par. 687) Hence he forms two indications in the cure of fevers, juſt according to thoſe diviſions for removing direct and indirect debility, (Elem. of M. par. 103—110) viz. In fevers dependent on direct debility, he recommends diffuſible ſtimuli in ſmall doſes, and often repeated; (par 686) in thoſe dependant on indirect debility, he orders the largeſt doſes of ſtimuli. (par. 687.)

In ſtating Dr. Darwin's Doctrine of Fever, the author premiſes a few obſervations or principles, viz.

"Before we enter into the conſideration of this doctrine, we ſhall lay down ſome principles deduced from the Zoonomia.

"1ſt. There is in every part of the animal ſyſtem a living principle, which is termed ſenſorial power, and which is conſidered as the immediate cauſe of all its motions (Zoon. part i. ſect. 4.) This is ſuppoſed to be ſecreted in the brain and ſpinal marrow, (Zoon. part i. ſect. 12, 2, 1.)

"2d. This living principle is capable of being acted upon in four different ways, viz. it poſſeſſes four different faculties or modes of action, which in their inactive ſtate are called irritability, ſenſibility, voluntarity, and aſſociability; and in their active ſtate, or while they are exerted, they are termed irritation, ſenſation, volition, and aſſociation. (Zoon. vol. i. ſect. 5.)

"3d. The faculty of that living principle, which is termed irritability, is exerted in conſequence of the ſtimulus of external bodies acting on any part of the ſyſtem where ſenſorial power reſides, and it then may produce fibrous motions. That of ſenſibility is exerted in conſequence of the ſtimulus of pleaſure or pain, occaſioned by the fibrous motions produced by the ſenſorial power of irritation at firſt. That of voluntarity is exerted in conſequence of the ſtimulus of deſire or averſion, occaſioned by the fibrous motions produced by the ſenſorial power of ſenſation at firſt. That of aſſociability is at firſt exerted in conſequence of the ſtimulus lus of any fibrous motions, previously occasioned either by irritation, sensation, or volition. (Zoon. vol. i. sect. 4 and 12.)

"4th. During the application of any of these stimuli, (Prin. 3d,) the living principle, or sensorial power, becomes exhausted; on the contrary, during the subduction of any of these stimuli the sensorial power becomes accumulated.

"5th. There are various circles of associate motions in the animal system, which may take their names from the nature of their introductory link: that is, those circles, the introductory link of which consists of an irritative motion, may be termed circles of irritative associate motions: those, the introductory link of which consists of a sensitive motion, circles of sensitive associate motions; and lastly, those, the introductory link of which consists of a voluntary motion, circles of voluntary associate motions. (Zoon. vol. ii. Class IV. 1, 1, B.)

"6th. The links of each of these circles act on one another by means of the sensorial power, which in its inactive state is called sensorial power of associability, and in its active state sensorial power of association. (Zoon. vol. i. sect. 5.)

"7th. These circles of associate motions may be affected by other sensorial motions, occasioned by the sensorial powers of irritability, sensibility, and voluntarity. (Zoon. vol. ii. Class IV. 1, 1, D.)

"8th. Each of these great circles of associate motions may be considered as compounded of smaller circles, that is, the great circle of irritative associate motions may be looked on as a collection of smaller circles of the same kind. (Zoon. Supl. 1st. 6.)

"9th. The introductory link of any circle of associate motions, may have its action increased, or decreased, or in its natural degree. The first may take place either in consequence of excess of sensorial power, the stimuli being in their accustomed degree; or, in consequence of excess of stimuli, the sensorial power being in its natural degree; or in consequence of excess of both. The second may arise either from want of sensorial power, the stimulus being in its usual degree; or from subduction of stimuli, the sensorial power being in its natural quantity; or from want of sensorial power, and subduction of stimuli. The third takes place, when both the sensorial power and the stimuli are in a proper degree.

"10th. Sometimes the morbid increased, as well as the morbid decreased, actions of the introductory link of a circle of associate motions are followed by similar actions of the other links; at other times by contrary actions: In the first case we have direct, in the second indirect sympathy. (Zoon. vol. i. sect. 35, 1; and vol. ii. Class IV. 1, 1, F.)

"11th. The morbid decreased actions, which arise from subduction of stimuli, are sooner overcome than those which are occasioned by want of sensorial power. (Zoon. vol. ii. Sup. 1, 12, 10.)

"12th. The morbid increased actions, which arise from excess of sensorial power, are more violent than those which are produced by by excefs of ftimuli. Hence inflammatory difeafes are commonly preceded by fubduction of ftimuli, and confequent accumulation of fenforial power, &c. But when excefs of fenforial power is acted upon by excefs of ftimuli, the exertion which follows is far fuperior. Hence mortification of frozen limbs, when brought near the fire. (Zoon. vol. ii. Clafs III. 2, 1, 17.)

"13th. Thofe parts which are fubjected, during health, to perpetual action, as the heart and arteries, accumulate fenforial power fafter, when their motions are impeded, than thofe which are fubjected to intermitted action. (Zoon. vol. ii. Supl. 1, 3, 1.)

"14th. When ftimuli, which are ufually applied to a certain part of the fyftem, are fubducted from it, an accumulation of fenforial power takes place there, proportioned to the fubduction of thofe ftimuli, and to the ftate of that part.

"15th. The exertion of any part of the fyftem may be either proper or greater, or fmaller than it ought to be; and fo either health, or inflammation, and the various degrees of exhauftion of fenforial power, or torpor from accumulation of fenforial power, will enfue.

"16th. Fever confifts of one or more difordered trains or tribes of affociated motions. (Zoon. vol. ii. Clafs II. 1, 2) Hence fever will be more or lefs complicated, according to the number of the tribes difordered.

"After thefe principles, Dr. Darwin's doctrine of fever may be confidered as follows: When the torpor of any part of the fyftem, owing to deficient irritation, occafioned either by the fubduction of the natural ftimuli, and confequent accumulation of fenforial power, or by the application of powerful ftimuli, and confequent exhauftion of the fame living principle (Prin. 4th, 7th, and 14th) is fuch as to occafion decreafed actions of that part, what happens? The next link of the tribe of affociate motions falls alfo into a torpor, from defect of excitement of the fenforial power of affociation, and fo the fubfequent one, till a general torpor affects the fyftem. This conftitutes the cold paroxyfm of fever. This general torpor remains, till the accumulation of the fenforial power of affociation has been formed, which may overbalance that defect of excitement of affociation, and then the torpor ceafes, and the hot fit of fever is produced.

"When the torpor of the part firft affected is occafioned by the fubduction of the natural ftimuli, this part is likewife thrown into increafed actions during the hot fit. But if it arife from exhauftion of fenforial power, this part remains in a torpid ftate during the hot fit. (Prin. 11)

"The torpor induced by the fubduction of the natural ftimuli, as it is overcome at the end of the cold fit, (§ XVIII) always gives rife to fevers with ftrong pulfe; fince in this cafe all the parts of the fyftem have their actions increafed during the hot fit. (Prin. 12)

"The

" The torpor induced by the exhauſtion of ſenſorial power produces various effects, according to the part in which it takes place. When it takes place in the ſtomach, it is always a cauſe of continued fever with weak pulſe. (Zoonomia, vol. ii. Supl. 1. 16. 9) In this caſe, in conſequence of the torpid ſtate of the ſtomach, the arterial ſyſtem falls likewiſe into a torpor, from defect of the excitement of the ſenſorial power of aſſociation; (Princ. 6 and 10) therefore an accumulation of this ſenſorial power of aſſociation takes place in the arterial ſyſtem: but this accumulation is ſo great, owing to the perpetual actions of the ſtomach catenated with thoſe of the arterial ſyſtem, (Princ. 13) that it affects the next link of the aſſociate train, that is, the capillaries, with increaſed energy. (Princ. 10) Hence theſe laſt, in this kind of fever, are perpetually exerted with great increaſe of action.

" When this torpor affects the ſecerning veſſels of the brain, Dr. Darwin thinks, that it is a cauſe of fever with arterial debility. (Zoonomia, vol. ii. Supl. 1. 10) In this caſe, the ſecretion of ſenſorial power being more or leſs impaired, muſt occaſion languid actions of every part of the ſyſtem. (Princ. 1) In fevers with arterial debility, ariſing from this cauſe, the actions of the capillaries are diminiſhed along with the actions of the reſt of the ſyſtem. Hence the heat of the body is never above the natural ſtandard, and ſometimes it is even lower throughout the courſe of the diſeaſe; which phenomenon I have obſerved ſometimes in patients labouring under continued fever with arterial debility.

" Though Dr. Darwin does not mention the torpor from exhauſtion of ſenſorial power in the arterial ſyſtem, as a cauſe of continued fever with arterial debility, yet, conſidering that the ſtimulus of the blood may be more or leſs increaſed, in conſequence of a greater or ſmaller quantity of oxygen, I make the following query: May not the ſenſorial power of irritation be accumulated for a while in the arterial ſyſtem, by the ſubduction of the ſtimulus oxygen? And afterwards, by a ſudden application of this ſtimulus in a great degree, that is, by inſpiring at once a large quantity of pure oxygen gas, may not a violent exertion take place there, ſo as to produce an exhauſtion of the ſenſorial power of irritation, and to render the arterial ſyſtem unfit to derive from the brain and ſpinal marrow a proper quantity of ſenſorial power, (See note (*l*) Princip. 11.) which may therefore give riſe to a fever with arterial debility?

[ To be continued. ]

MONTHLY

